# Increasing response rates from physicians in oncology research: a structured literature review and data from a recent physician survey

**DOI:** 10.1038/bjc.2012.28

**Published:** 2012-02-28

**Authors:** Y Martins, R I Lederman, C L Lowenstein, S Joffe, B A Neville, B T Hastings, G A Abel

**Affiliations:** 1 Center for Population Sciences, Dana-Farber Cancer Institute, Boston, MA, USA; 2 Department of Medicine, Children's Hospital Boston, Boston, MA, USA; 3 Department of Pediatric Oncology, Dana-Farber Cancer Institute, Boston, MA, USA; 4 Department of Medical Oncology, Dana-Farber Cancer Institute, 450 Brookline Avenue, Dana 1108, Boston, MA 02215, USA

**Keywords:** surveys, response rates, health services research, oncology

## Abstract

Although the physician survey has become an important tool for oncology-focused health services research, such surveys often achieve low response rates. This mini-review reports the results of a structured review of the literature relating to increasing response rates for physician surveys, as well as our own experience from a survey of physicians as to their referral practices for suspected haematologic malignancy in the United States. PubMed and PsychINFO databases were used to identify methodological articles assessing factors that influence response rates for physician surveys; the results were tabulated and reviewed for trends. We also analysed the impact of a follow-up telephone call by a physician investigator to initial non-responders in our own mailed physician survey, comparing the characteristics of those who responded before *vs* after the call. The systematic review suggested that monetary incentives and paper (*vs* web or email) surveys increase response rates. In our own survey, follow-up telephone calls increased the response rate from 43.7% to 70.5%, with little discernible difference in the characteristics of early *vs* later responders. We conclude that in addition to monetary incentives and paper surveys, physician-to-physician follow-up telephone calls are an effective method to increase response rates in oncology-focused physician surveys.

Although surveys of practicing physicians are a valuable source of data to help guide health care policy, they typically have poor response rates (Cummings *et al*, 2001; Kellerman and Herold, 2001; VanGeest *et al*, 2007). Specifically, the average response rate of physicians to mailed surveys has traditionally been demonstrated to be only 54% to 58% (Martin, 1974; Asch *et al*, 1997; Cook *et al*, 2009) – 14% lower than that of non-physicians – and appears to be getting worse in the era of modern multimedia communications (Cull *et al*, 2005; Cook *et al*, 2009). In addition to their potential impact on population-based health care decisions, physician surveys often form the cornerstone of quality improvement efforts, as such efforts cannot take place without a reliable assessment of providers’ current practices and attitudes. If physician non-response leads to survey bias, resulting policy and practice decisions may not accurately represent the views and practices of the target population being sampled.

In oncology, as in other areas of medicine, physician surveys are increasingly being undertaken to assess patterns and quality of cancer care. Recent oncology-related physician surveys published in high-profile journals include an assessment of attitudes of American *vs* Canadian oncologists as to the cost-effectiveness of new cancer drugs (response rate 59% (Berry *et al*, 2010)), primary care physicians’ (PCPs’)(Del Giudice *et al*, 2009) and oncologists’ (Greenfield *et al*, 2009) views of appropriate follow-up care for cancer survivors (response rate 52% for the former and 36% for the latter), an assessment of surgeons’, medical oncologists’ and radiation oncologists’ involvement in clinical trials (response rate 61% (Klabunde *et al*, 2011)) and a survey of oncologists’ views regarding communicating the costs of chemotherapy to patients (response rate 31.5% (Schrag and Hanger, 2007)). As can be seen, physician response rates in these studies vary widely; clearly, those with higher response rates have the potential to be much more influential in informing these diverse areas of cancer-related health care policy.

In their comprehensive literature review, Edwards *et al* (2009) found that successful methods for increasing response rates to postal surveys include monetary incentives, use of shorter questionnaires, follow-up contact, and reply envelopes that contain a stamp rather than metered postage. Responses to electronic surveys were found to be increased with non-monetary incentives, shorter surveys, a lottery with instant notification of results, and exclusion of the word ‘survey’ in the email invitation subject line. Although Edwards *et al* (2009) analysis is informative, <10% of the 513 survey studies reviewed included physician respondents. Indeed, relatively few studies have specifically examined strategies to increase physicians’ responses to surveys (Field *et al*, 2002), and the last major review of the literature was published in 2007 (VanGeest *et al*, 2007).

Our goal was to review the current literature relating to obtaining high physician-survey response rates, with an eye towards improving such efforts in oncology-related research. We then aimed to present our own experience increasing the response rate of a survey targeting primary care physician behaviours with respect to referral for suspected haematologic cancers. We specifically desired to present our data to an audience of clinicians and investigators likely to undertake such surveys as part of their clinical quality improvement efforts or cancer-related health services research.

## Methods

### Structured literature review

English language experimental studies and literature reviews of methods to improve physician response rates were identified through searches of PubMed and PsycINFO databases, focusing on the years 2000 to 2010. We chose this time frame because we felt that prior reviews had sufficiently examined older work, and because we wanted to focus our analysis on more recent studies that would be most likely to assess both postal and electronic approaches.

Keywords used in binary combinations included: ‘physician survey’, ‘response rate’, ‘improved’, ‘questionnaire’, ‘incentives’, ‘Internet’, ‘web’, ‘mail’, and ‘postal’. Eight prior review articles regarding physician surveys (Cummings *et al*, 2001; Kellerman and Herold, 2001; McColl *et al*, 2001; Field *et al*, 2002; Braithwaite *et al*, 2003; Cull *et al*, 2005; VanGeest *et al*, 2007; Cook *et al*, 2009) were also assessed to identify additional primary papers. A review of relevant abstracts of primary and secondary searches revealed 38 that focused on experimental studies specifically examining factors that affect physician response rates; the full texts of these were obtained for further review (Table 1). Despite the fact that many surveys of physicians both within and outside of cancer medicine have achieved excellent response rates, we rejected articles that did not specifically compare methods for improving response rates using the same survey and physician sample. We did so because we did not feel we could rigorously compare the methods used across different analyses, given the many disparate topics and samples studied.

### Physician survey case study

From April to August 2010, we surveyed PCPs in Massachusetts regarding their practice patterns with respect to the diagnosis and referral of patients with suspected haematologic malignancy. Our survey was designed to detrmine the approximate number of patients seen in the last year that PCPs suspected might have haematologic malignancy, the frequency of formal specialty referral for those patients, and the frequency of informal curbside consultation. PCPs were also queried about the factors that influence their choice of specialist, and about the information exchange with the specialist.

The names of 6836 Massachusetts physicians were obtained from the American Medical Association; 375 of these were randomly selected for inclusion in the survey. We then searched the Massachusetts Board of Registration in Medicine online directory to verify that the physicians met the study's eligibility criteria: (1) currently in practice at Massachusetts; (2) graduated from medical school in 2005 or earlier; (3) listed specialty or board-certified in internal medicine, general medicine, family medicine or geriatrics; and (4) no non-primary care subspecialty listed. Investigating each name on the initial list took approximately 3.6 min, for a total of 22.5 h spent on cleaning procedures. The final pre-contact eligible sample consisted of 250 physicians. Of these, 60 reported upon contact that they did not engage in primary care and were reclassified as ineligible. The final eligible sample included 190 PCPs.

#### Initial recruitment

Each physician received a package delivered to her/his office using FedEx courier services, identifying the study physician-investigator (GAA) as the sender. The package included a letter inviting the physician to participate, a printed survey, opt-out card, and a pre-paid, self-addressed return envelope. The letter directed participants to either fill out and return the paper survey, or log-on to a secure website to complete the survey over the Internet. The opt-out card allowed physicians to report that they either declined to participate or that they were ineligible because they did not engage in primary care. Reminder postcards were sent to those physicians who had not yet completed the survey 2 weeks later. Three weeks after that, a second package containing the same materials and instructions was sent to all physicians who had not responded. Physicians who responded after these first three solicitations were termed ‘early responders’.

#### Telephone recruitment

Telephone calls were made to each physician who had not yet responded by the study's principal investigator (GAA), a medical oncologist, 7 weeks after the initial package was sent. If a potential physician respondent was not available, the study physician either left a message asking for his call to be returned, or, if directed to a voicemail system, a more detailed message regarding the survey itself. Potential physician respondents who were not reached during the first round of telephone calls were called again approximately 2–3 weeks after the initial call. Physicians who responded after the telephone calls were termed ‘late responders’. Regardless of recruitment methodology, those who completed the survey received a $100 VISA gift card by mail.

#### Analysis

After recruitment was complete, we assessed the overall response rate, as well response rates before and after the follow-up telephone calls. Next, using *χ*^2^ analysis or the Fischer's Exact test depending upon on how many subjects were available for each category, we analysed whether there were differences in self-reported characteristics among early *vs* late responders (gender, age, race, ethnicity, years post residency and practice type) and whether there were difference in characteristics obtained from the Massachusetts Board of Registration in Medicine website (gender, practice type, medical school location and years since graduation) among responders *vs* non-responders.

## Results

### Structured literature review

We found that studies of physician response rates generally have tested the effects of the mode of survey, type of incentive, or other interventions (Table 1). The interventions examined varied greatly, but monetary incentives were generally effective (9/11 positive studies), and paper surveys engendered more responses than surveys delivered in other formats such as email (7/8 positive studies). Interestingly, one study demonstrated that response rates were even better with a mailed survey that had an option to respond by email, a so-called ‘mixed methods’ approach (Seguin *et al*, 2004).

When using an incentive, the studies suggested that it is better to ‘pre-pay’ by sending the incentive with the survey itself *vs* ‘post-pay’ after completion (Leung *et al*, 2002), and that cash is preferable to a gift (such as a pen (Clark *et al*, 2001b)). In addition, a personalised cover letter stressing the importance of that individual physician's reply was shown to result in a better response rate (Leece *et al*, 2006). Data on the use of enrollment in a lottery as an incentive was more complex. One study suggested that enrollment in a lottery in exchange for completing a survey ($500 Canadian) was better than nothing at all (Baron *et al*, 2001), but another found that even a small incentive given to all ($2 US upfront) was better than the chance of enrollment in a lottery for a bigger prize ($250; Tamayo-Sarver and Baker, 2004).

Interestingly, some factors that one might assume would lead to a better response rate did not always help and could even be detrimental; for example, one study demonstrated that the addition of a letter featuring the endorsement of the survey by an expert lead to significantly lower primary response rates (Bhandari *et al*, 2003). Other factors that may have a positive effect included shorter survey word length (Jepson *et al*, 2005) and inclusion of a stamped return envelope *vs* a business return envelope (Streiff *et al*, 2001). This final analysis was the only one to present methodological data from a study of haematologists or oncologists (a mailed survey of 3000 members of the American Society of Hematology to assess their approach to diagnosis and treatment of polycythemia vera; response rate was 38% with the stamped envelope).

### Physician survey case study

In our own survey, follow-up telephone calls from the physician investigator increased physician response rates from 43.7% to 70.5%. In total, these phone calls took approximately 20 h, for an average of 23.5 min of physician effort required to recruit each additional participant. Early and late responders did not differ in age, race, ethnicity, years since residency or practice type (Table 2; all *P*>0*.05*). In contrast, female physicians were more likely to be early responders (*P*<0.01, Table 2).

Comparing responders to non-responders, we found similar proportions trained in foreign medical schools (24% for responders *vs* 27% for non-responders; *χ*^2^=0.17, *ns*) and a similar distribution among the two groups of family medicine, general practice, and internal medicine practices (*χ*^2^=4.45, *ns*). The proportions of males and females were reversed between responders and non-responders (*χ*^2^=6.62, *P*<0.*05*) such that 40% of responders were female and 60% were male, whereas non-responders were 61% female and 39% male. Finally, those who had graduated from medical school within the past 10 years were significantly more likely to respond (91%) than those who graduated more than 11 years before (65% to 67% across for those 11 to 20 years, 21 to 30 years, or 31+ years post graduation; *χ*^2^=8.02, *P*<0.*05*).

## Discussion

Our literature review revealed that several factors have the potential to increase response rates to physician surveys, such as the inclusion of monetary incentives and the use of paper *vs* web or email formats. Several other items – from shorter survey word length to the use of a personalised cover letter – were also demonstrated to help. In addition, our case study suggested that telephone calls made by a physician investigator to potential physician respondents may greatly increase response rates among initial non-responders.

We found little difference between early and late responders with respect to most socio-demographic dimensions in our survey. This finding is reassuring, as it suggests that medical peer follow-up calls may not greatly change the characteristics of those who ultimately respond. On the other hand, although effective, personal calls by physician researchers are costly, and unlikely to be feasible for the large samples that are sometimes encountered in oncology-related health services research. Unfortunately, whether or not a follow-up telephone call by a research assistant or other non-peer clinician can capture some of that benefit with respect to increasing response rates remains unclear.

Two older studies (before 2000 so not included in our literature review above) assessed the effect of direct follow-up contact from a medical peer on physician survey response rates. The first found that follow-up telephone calls by investigating physicians to PCPs improved response rates from 62% to 92% (Bostick *et al*, 1992). In the other – a study of PCPs regarding their oncology consultation practices – response rates were increased from 44% to 78% after follow-up telephone calls from a medical peer to initial non-responders (Heywood *et al*, 1995). Our case study demonstrated a slightly smaller increase in response rate (27% *vs* 30% and 34% in the before studies); however, we may conclude that despite the modern milieu of email, text messaging, and social media, a follow-up peer-to-peer telephone call still has an important role in terms of assuring high physician response rates. Our results also correspond to the broader survey literature that suggests follow-up contact is essential (Edwards *et al*, 2009).

We found that overall, respondents (early and late) were more likely to be recent graduates and also to be male. Although the former finding is consistent with prior studies – perhaps because as more recent licensees, specialty and contact information for younger graduates obtained from public sources is more likely to be accurate (Kellerman and Herold, 2001; Barclay *et al*, 2002; Cull *et al*, 2005) – these same studies have shown that female physicians are generally more likely to respond. On the other hand, our own gender results are consistent with another large survey that used the American Medical Association physician file (McFarlane *et al*, 2007), which suggests that our source of respondents may have had a role.

Other than one analysis (Streiff *et al*, 2001), we found no other examples of methodological studies specifically assessing how to increase response rates for surveys of oncology specialty physicians. Although oncology-related surveys of PCPs can make use of the general literature on surveying physicians (as we ourselves did in our case example), additional strategies may be important to increase response rates from oncology specialists. With respect to the latter, empirical research is clearly needed (e.g., focus groups, key informant interviews or even surveys of oncologists). Possible strategies that may emerge include having the survey endorsed by an oncology specialty society (ours was not) or administered at a national oncology meeting (ours was not). Still, it may be that a ‘one size fits all’ strategy will not be the answer in oncology, and that tailoring the approach to the specific target physician population and investigative aims will dictate the best method.

Our own survey experience illustrates the importance of using a ‘clean’ sample, where attempts at verification of eligibility are made before contacting potential respondents. Indeed, despite our extensive efforts, we still contacted some physicians that were ultimately ineligible. Although such sample cleaning is time consuming and expensive, it is necessary to ensure that a pool of respondents representative of the target population is obtained, irrespective of the sample size. In addition, this can help the ultimate response rate, because, when an ineligible physician does not respond, unless that status is confirmed, he or she must be included in the response rate denominator, which has the effect of lowering the ultimate response rate. Our study also speaks to the utility of the mixed-methods approach (both postal and electronic options for reply), which may be the best way to obtain a high response rate from physicians (Beebe *et al*, 2007; Sprague *et al*, 2009) especially as Internet-only (Leece *et al*, 2004) and email-only (McMahon *et al*, 2003) approaches have been suffering from lower response rates compared with mailed surveys.

We recognise limitations to our work. First, it is conceivable that some analyses of factors that impact physician survey response rates may have been missed in our structured literature review. Indeed, our search terms were broad, and deciding which studies to include as primarily ‘methodological’ was necessarily subjective. Second, in our case study, the principal investigator was an oncologist telephoning PCPs, and it may be that increases in response rates would have differed if he were also a PCP (possibly better) or if he were telephoning fellow oncologists (possibly better). Third, it was not possible to determine whether the difference in response rates found between early and late responders in our case study was statistically significant (although the magnitude of the difference suggests it was), because the latter group included the former, and thus the two were not independent groups for which there is an appropriate statistical test. Finally, Massachusetts is a state with universal health care and a dense network of hospitals and physicians. Certainly, follow-up calls from a study physician may have different effects on physician response rates in states or countries with a different health care environment.

In summary, as the landscape of clinical practice, health insurance and health care policy evolves, it is likely that physicians will be solicited more often to complete surveys. The use of survey methods that include physicians will also likely increase in cancer medicine, a field with many health services issues ripe for study using such methods. Our work results in several recommendations for the oncology-focused physician survey. First, using a mailed survey (usually by a courier company such as FedEx) makes sense, with an option to be filled out via email or Internet. Second, we recommend a personalised letter including an upfront monetary incentive if possible. Third, paying attention to details such as shorter survey word length and stamped returned postage *vs* business reply envelope may be important. Finally, follow-up contact should proceed on a regular schedule, and a follow-up call by a peer physician-investigator, when feasible, may be a particularly effective tool.

## Figures and Tables

**Table 1:** Studies assessing interventions to improve physician response rates, 2000–2010*

**Study**	**Intervention(s)**	**Results**	**Significance**
*Mode of survey*			
Beebe *et al* (2007)	Web w/mail follow-up *vs* mail w/web follow-up	Web 1^st^:62.9% mail 1^st^: 70.5%	NS
Grava-Gubins and Scott (2008)	(1) Long *vs* short survey (2) Post *vs* email	(1) Long: 31.7% short: 31.6% (2) Post: 34.1% email: 29.9%	(1) NS (2) NS
Hocking *et al* (2006)	Post *vs* phone survey	Post: 59.9% phone: 40.6%	RR=1.5 (1.3–1.7)
Leece *et al* (2004)	Web *vs* paper	Web: 45% paper: 58%	*P*<0.01
Lensing *et al* (2000)	Choice of fax, telephone or mail questionnaire	Requests – fax: 47% phone: 28% mail: 25%	No test
McMahon *et al* (2003)	Email *vs* fax *vs* post, two contacts	Fax: 47% post: 41% email: 26%	No test
Raziano *et al* (2001)	Web *vs* post	Web: 58% post: 77%	*P*< 0.01
Seguin *et al* (2004)	Email *vs* post with email access *vs* post only	Email: 33.6% post with email access: 52.7% post only: 47.8%	No test
VanDenKerkhof *et al* (2004)	Web *vs* post	Web: 35% post: 69%	RR=0.51 (0.45–0.58)
			
*Incentives*			
Baron *et al* (2001)	Lottery *vs* no lottery	Lottery: 41.2% no lottery: 34.8%	*P*< .05
Burt (2003)	(1) Motivational insert *vs* control (2) Cash *vs* token gift *vs* control (3) Short form *vs* long form	(1) Insert: 68.2% control: 64.3% (2) Cash: 72.7% gift: 67.6% control: 72.7% (3) Short: 67.6% long: 61.9%	(1) NS (2) No test (3) *P*< 0.05
Clark *et al* (2001b)	Pen *vs* no pen	Pen: 69% no pen: 68%	NS
Delnevo *et al* (2004)	Pre-payment *vs* post-payment	Pre-pay: 71.5% post-pay: 56%	*P*<0.01
Gattellari and Ward (2001)	Promised a donation *vs* not	Donation: 84.3% nothing promised: 93.7%	*P*<0.05
Halpern *et al* (2002)	(1) $5 *vs* $10 (2) Large envelope *vs* small	(1) $5: 52.8% $10: 60.5% (2) Large: 55.5% small 56.6%	(1) *P*<0.01 (2)NS
Keating *et al* (2008)	$20 *vs* $50	$20: 52.1% $50: 67.8%	*P*<0.01
Leung *et al* (2002)	(1) No Incentive *vs* incentive (2) Cash *vs* lottery	(1) Incentive: 19.8% no incentive: 16.8% (2) Cash: 27.3% lottery: 19.4%	(1) *P*<0.02 (2) *P*<0.02
Leung *et al* (2004)	Pre-payment *vs* post-payment	Pre-pay: 82.9% post-pay: 72.5%	*P*<0.01
McDermott *et al* (2003)	Pre-payment *vs* post-payment incentive (CME credits and $5)	Non-surgeons, Pre-pay: 70.7% post-pay: 83.2% Surgeons, pre-pay: 98.9% post-pay: 95.6%	No test
Moses and Clark (2004)	Prize drawing *vs* not in drawing	Offered prize: 64% no prize: 62%	NS
Puleo *et al* (2002)	(1) 1st class mail + coffee, (2) 1st class mail non-responders, (3) Phone calls or email by known names (4) Telephone survey attempt	Cumulative response rates: (1) 40% (2) 64% (3) 74% (4) 91%	No test
Recklitis *et al* (2009)	Non-monetary *vs* monetary *vs* both	Non-monetary: 63.0% monetary 81.6% both 76.3%	*P*<0.01
Robertson *et al* (2005)	Incentive *vs* no incentive	Incentive: 49.7% no incentive: 40.1%	*P*<0.01
Tamayo-Sarver and Baker (2004)	$2 in mailing *vs* lottery for $250	$2: 56% lottery: 44%	*P*<0.01
Thomson *et al* (2004)	Lottery for one big prize *vs* many small prizes	One big prize: 68% many small prizes: 59%	*P*<0.05
Thorpe *et al* (2009)	No incentive *vs* incentives and/or recorded delivery/registered mail	No incentive : 48% other strategies: 74%–76%	No test
VanGeest *et al* (2001)	$5 *vs* $10 *vs* $20	$5: 60.3% $10: 68% $20: 65.2%	NS
			
*Other variables*			
Barclay *et al* (2002)	Timing of follow-up in a mailed survey: Day 0, 11, 20, 61	Day 0: 36.9% Day 11: +14.9% Day 20: +11.4% Day 60: +4.4% total response rate=67.6%	No test
Bergk *et al* (2005)	Follow-up in a mailed survey: (1) Questionnaire (2) Reminder (3) Reminder+2nd copy	(1) 33% (2) +6.8% (3) +18.4%	No test
Bhandari *et al* (2003)	Endorsement letter *vs* not	Endorsement: 47.5% no endorsement: 59.8%	*P*<0.05
Brehaut *et al* (2006)	(1) Single *vs* double-sided printing (2) Known *vs* unknown sender	(1) Single: 73.2% double: 65.8% (2) Known: 73.6; unknown: 66.3%	(1) NS (2) NS
Clark *et al* (2001a)	High *vs* standard paper quality	High: 22% standard: 29%	NS
Drummond *et al* (2008)	(1) Demographics first *vs* Demographics later (2) Pre-contact *vs* no Pre-contact	(1) Demographics first: 50.6% later: 45.4% (2) Pre-contact: 49.8% no pre-contact: 46.2%	(1) *P*=0.05 (2) NS
			
Jepson *et al* (2005)	Surveys of various word lengths	>1000 words: 38% <1000 words: 59.4%	*P*<0.01
Jiwa *et al* (2004)	Post with reminders *vs* given at educational meeting	Post: 76% meeting: 83%	*P*⩽0.01
Leece *et al* (2006)	Standard cover letter *vs* personalised cover for both post and email	Post: standard cover: 30% personalised cover: 47% Email: standard cover: 23% personalised cover 22%	(1) *P*<0.01 (2) NS
McKenzie-McHarg *et al* (2005)	Hand-signed *vs* computer-signed	Hand-signed: 79.1% computer-signed: 78.4%	NS
Streiff *et al* (2001)	Stamped return postage *vs* business postage	Stamped: 38% business postage: 32%	*P*<0.01

Abbreviation: NS=not significant.

% reported are response rates unless otherwise specified.

* As of September 1, 2010.

**Table 2:** Percentage (number) of early and late responders as a function of demographic variables in a survey of PCPs as to referral patterns for haematologic malignancy^a^

**Variable**	**Test**	**Early responder**	**Late responder**	***P***
*Gender*				
Female	*χ* ^2^	49 (26)	51 (27)	<0.01
Male		74 (58)	26 (20)	
				
Age^b^	*t*	48.3	47.2	0.6
				
*Race*				
Asian	Fisher's exact	65 (17)	35 (19)	0.3
White		66 (61)	34 (32)	
Black		25 (1)	75 (3)	
AI/AN		100 (1)	0 (0)	
Other		50 (4)	50 (4)	
Multi-racial		0 (0)	100 (1)	
				
*Ethnicity*				
Hispanic	Fisher's exact	75 (6)	25 (2)	0.7
Non-Hispanic		63 (78)	37 (45)	
				
Years post residency^b^	*t*	16.4	15.9	0.8
				
*Practice type*				
Family	Fisher's exact	69 (27)	31 (12)	0.5
Internal		61 (51)	39 (32)	
Other		0 (0)	100 (1)	
Multiple		56 (5)	44 (4)	

Abbreviations: AI=American Indian; AN=Alaska Native; Family=family practice; Internal=Internal Medicine; Multiple=respondent selected more than one practice type; Other=all other practice types; PCP=primary care physician.

a Total numbers for each variable differ due to differing numbers of respondents answering each question and/or more than one response.

b Data presented are means.
